# Known and novel parvoviruses identified in domestic pigeons

**DOI:** 10.1186/s12917-025-04510-8

**Published:** 2025-01-31

**Authors:** Ewa Łukaszuk, Daria Dziewulska, Anthony Khalifeh, Joy M. Custer, Simona Kraberger, Arvind Varsani, Tomasz Stenzel

**Affiliations:** 1 Department of Poultry Diseases, Faculty of Veterinary Medicine, University of Warmia and Mazury in Olsztyn, Olsztyn, Poland; 2 Biodesign Center for Fundamental and Applied Microbiomics, Center for Evolution and Medicine, School of Life Sciences, Arizona State University, Tempe, USA; 3 Structural Biology Research Unit, Department of Integrative Biomedical Sciences, University of Cape Town, Observatory, Cape Town, South Africa

**Keywords:** *Columbidae*, Coding-complete genome, Digital droplet PCR, Enteropathy, High-throughput sequencing, *Parvoviridae*, Pigeon, SsDNA virus

## Abstract

**Background:**

*Parvoviridae* is a family of single-stranded linear DNA viruses whose members infect both vertebrate and invertebrate species of animals, causing diseases of various systems and often associated with pathology of the gastrointestinal tract. Additionally, parvoviruses are known to induce illnesses causing diarrhea in various avian species as well; however, data on their occurrence and pathology in pigeons is scarce.

**Results:**

In this study, we developed molecular biology methods to detect and quantify parvovirus genetic material in samples acquired from racing pigeons of different health status. Our intention was to determine a connection between the presence of the virus and the occurrence of clinical signs in sampled birds. The results of quantitative analysis indicate no direct association of parvoviruses with the manifestation of enteric disease in pigeons. High-throughput sequencing was performed on samples testing positive in quantitative PCR with TaqMan probe and in digital droplet PCR. It allowed us to assemble two coding-complete pigeon parvovirus genomes, one belonging to new species and referred to as pigeon parvovirus 2, and the second which is a member of species *Aveparvovirus columbid1*. Additionally, we analyze two coding-complete genomic sequences acquired from pigeon feces in USA, one representing species *Aveparvovirus columbid1* and one being a member of *Chaphamaparvovirus* genus in *Hamaparvovirinae* subfamily.

**Conclusions:**

This is the first report of parvovirus in pigeons outside Asia. The findings of our research emphasize the need to further explore the poorly understood biology and pathology of pigeon parvoviruses.

**Supplementary Information:**

The online version contains supplementary material available at 10.1186/s12917-025-04510-8.

## Background

Members of family *Parvoviridae* are non-enveloped viruses of 23–28 nm in diameter, possessing linear, non-segmented, single-stranded DNA genomes of 4–6 kb which typically encode two proteins: a non-structural protein (NS1) which is the replication initiator protein and a structural protein (VP) which is the viral protein forming the capsid; some parvoviruses also encode other minor proteins [1]. Amino acid sequence of NS1 is used for species demarcation—members of the same species share > 85% amino acid identity [2]. The family is currently comprised of over 180 species, divided into three subfamilies: *Densovirinae* subfamily which consists of viruses infecting invertebrates, *Parvovirinae* subfamily which consists of viruses infecting vertebrates, and *Hamaparvovirinae* subfamily including both viruses of invertebrates and vertebrates [3]. Parvoviruses are common in humans and various animal species, and they are often associated with gastrointestinal illness presenting with diarrhea, but also can be a cause of diseases affecting many other systems, including respiratory, immune or reproductive system [4–11].

Many parvoviruses have also been identified in birds – members of the *Aveparvovirus* and *Dependoparvovirus* genera of *Parvovirinae* subfamily and the *Chaphamaparvovirus* genus of *Hamaparvovirinae* subfamily have been detected in several domestic and wild avian species, with genus *Aveparvovirus* consisting of viruses for which birds are the only known hosts [12–18]. Some diseases caused by viruses in the *Parvovirinae* subfamily are well studied, e.g., Derzsy’s disease caused by Muscovy duck parvovirus and Barbarie duck parvovirus of the species *Dependoparvovirus anseriform1,* which is an illness of high morbidity and mortality affecting goslings and young Muscovy ducks [19, 20]. Another common health problem connected with parvoviral infection is runting-stunting syndrome causing diarrhea, impaired growth and poor feathering in chickens. Chicken parvovirus of the species *Aveparvovirus galliform1* is known to play a role in this syndrome, among other viruses [21–23]. Parvoviruses can also infect pigeons, however, not much is known about the impact of these viruses on pigeon health. To date, only one paper describing the finding of genetic material of pigeon parvovirus classified in the species *Aveparvovirus columbid1* in samples collected from pigeons has been published [12]. Therefore we made efforts to introduce methods that would help to uncover a part of this poorly explored topic.

In this study, we developed molecular approaches to detect and quantify parvovirus genetic material in samples acquired from racing pigeons. Our intention was to investigate possible associations with parvoviral infection and disease symptoms in sampled birds. In addition, we identified new and known pigeon-associated parvoviruses and used phylogenetic analysis to explore the diversity of parvoviruses occurring in pigeons.

## Methods

### Sample collection

Cloacal swab samples were collected in 2023 from racing pigeons originating from lofts located in various parts of Poland, and there were no connection between the birds originating from individual examined lofts. The selection of birds for the study has been described in detail in our previous studies [24, 25]. 90 pigeons from 24 flocks exhibiting signs of enteropathy were selected for the study group (S) and 63 pigeons from 13 healthy flocks were selected for the control group (C). The cloacal swabs collected from birds from each group were suspended in the UTM® Universal Transport Medium (Copan Diagnostics, Murrieta, California, USA), which was then aliquoted in two portions and stored at −80 °C until testing.

As a part of an ongoing avian-infecting viral diversity study we opportunistically collected four fecal samples from feral pigeons of unknown health status in Tempe, Arizona (USA) on the 13 January 2021.

### Extraction of DNA

The first part of the transport medium of swab samples from Poland was used for DNA extraction, which was conducted using the DNeasy Blood & Tissue Kit (Qiagen, Hilden, Germany) according to the manufacturer's protocol. The extracted DNA was eluted in 50 μl of elution buffer, purity and concentration were assessed with a NanoDrop 2000 Spectrophotometer (Thermo Fisher Scientific, Waltham, Massachusetts, USA) and the samples were stored at −80 °C until further testing.

For the fecal samples collected in USA, ~ 5 g of each sample was homogenized in 2 ml of SM buffer. The homogenate was then sequentially filtered through 0.45 and 0.2 µm syringe filters, and 200 µl of the filtrate was used for viral DNA extraction using the High Pure Viral Nucleic Acid Kit (Roche, Basel, Switzerland).

### Quantitative analysis

#### TaqMan qPCR

A novel quantitative PCR assay with TaqMan probe (TaqMan qPCR), targeting a 143 bp fragment of non-structural protein gene, was developed to test the samples for the presence of genetic material of pigeon parvovirus 1 and to assess Cq values. It was conducted as a first part of quantitative analysis, because samples with Cq lower than 26 might present as oversaturated in the next step which is digital droplet PCR (ddPCR). This precheck allowed identification and appropriate dilution of such samples before the ddPCR assay. Primers (forward: 5ꞌ-CCTCATGCTCAACTCTGCGA-3ꞌ and reverse: 5ꞌ-TAGAGGTCCTTCTCGCTGCT-3ꞌ) and probe (5ꞌ-[6-FAM]TGAACACACCGTCGAGCTG [BHQ-1]−3ꞌ) for the assay were designed with ‘Design New Primers’ tool available in Geneious Prime v. 2024.0.3 software (Dotmatics, Boston, Massachusetts). The pigeon parvovirus 1 sequences used to design the primers and the probe were either downloaded from the NCBI GenBank database (KC876004, OK376644 and OR800323) or originating from our collection (PP965732).

The reaction mixture was composed of 10 µl of TaqMan™ Fast Universal PCR Master Mix kit (Thermo Fisher Scientific, Waltham, Massachusetts), 1.8 μl of both 10 μM primers, 2 μl of 2.5 μM probe, 1.4 µl of nuclease-free water and 3 µl of DNA. The qPCR reaction was conducted in the LightCycler® 96 System thermocycler (Roche, Basel, Switzerland) and included 30 s of preincubation at 95 °C and 40 cycles of two-step amplification at 95 °C for 15 s and at 58 °C for 40 s.

Prior to screening of the samples collected for the study, the specificity of the assay had been tested using the genetic material of known pigeon viruses such as pigeon adenovirus 1, pigeon circovirus and pigeon herpesvirus, originating from the collection of Department of Poultry Diseases, Faculty of Veterinary Medicine, University of Warmia and Mazury in Olsztyn, Poland, as a template for the reaction. Then, the sensitivity of the assay was assessed by amplifying a 921 bp fragment of non-structural protein gene, overlapping with the fragment amplified in qPCR. The reaction mixture was made by mixing 10 μl of HotStart TaqPlus DNA Polymerase (Qiagen, Hilden, Germany), 0.1 μl of 100 μM primers (forward: 5ꞌ-GTGGTGGGAGGAATGCATCA-3ꞌ and reverse: 5ꞌ-CTCCTCCATCAGACCCTCGT-3ꞌ) designed using the same sequences as in qPCR, 6.8 μl of nuclease-free water and 3 μl of DNA, and the reaction was conducted in a Mastercycler thermal cycler (Eppendorf, Hamburg, Germany). The conditions were as follows: 95 °C for 5 min, 40 cycles of 94 °C for 30 s, 56 °C for 1 min and 72 °C for 1 min, and 72 °C for 10 min. The amplicons were cleaned up with a Clean-Up kit (A&A Biotechnology, Poland), the concentration and purity were assessed with a NanoDrop 2000 Spectrophotometer and the gene copy number was calculated with a copy number calculator (Genomics and Sequencing Center, University of Rhode Island, Kingston, Rhode Island). The standard curve was plotted using tenfold serial dilutions from 3.018 × 10^7^ to 3.018 copies of the amplicon/μl, and the reactions were performed in triplicate.

Finally, the samples analyzed in this study were assayed in duplicate, and pigeon parvovirus 1 sample archived in the laboratory of Department of Poultry Diseases (Faculty of Veterinary Medicine, University of Warmia and Mazury in Olsztyn, Poland) served as a positive control. Samples with Cq value lower than 36 were considered positive and prior to ddPCR run, those with Cq of 25 or lower were diluted with nuclease-free water according to the principle that one tenfold dilution increases Cq by 3.

#### ddPCR

ddPCR assay was developed to allow absolute quantification of pigeon parvovirus 1 genetic material in samples positive in qPCR. The primers, probe and positive control were the same as in qPCR and all samples were analyzed in duplicate. The reaction mixture consisted of 11 µl of ddPCR Supermix for Probes (Bio-Rad, Hercules, California, USA), 1.98 µl of primers and 2.2 µl of probe, 1.84 µl of RNase-free water and 3 µl of DNA. The generation of droplet emulsions, in which the reaction would be conducted, proceeded as described in our previous studies [24, 25]. The PCR amplification was performed with C1000 Touch Thermal Cycler (Bio-Rad, Hercules, California, USA) under the following conditions: 10 min of preincubation in 95 °C, 40 cycles of two-step amplification in 94 °C for 30 s and in 52 °C for 1 min, and 4 °C for 30 min. All steps had a ramp rate of 2 °C/s. Then, a QX 200 Droplet Reader (Bio-Rad, Hercules, California, USA) was used to assess the number of viral amplicons in each sample. The results were presented as mean number of pigeon parvovirus 1 amplicon copies ± standard deviation per 20 µl of the sample, while the values for previously diluted samples were multiplied by the dilution value.

### High-throughput sequencing

The second part of the transport medium of samples that tested positive in qPCR was used for the high-throughput sequencing (HTS). DNA was extracted from the samples and used as a template for rolling-circle amplification (RCA) using Phi29 enzyme kit (Watchmaker Genomics, USA), dNTPs (Thermo Fisher Scientific, USA) and random hexamers (Integrated DNA Technologies, USA) to enrich the samples with circular molecules. The RCA was performed according to the instructions of the Phi29 enzyme kit manufacturer. The resulting amplicons were pooled per sample with the original DNA extract at a 50:50 ratio. These were then used to generate Illumina sequencing libraries using the TruSeq Nano DNA Kit with ~ 350 bp inserts. These multiplexed libraries were quality checked and sequenced (2 × 150 bp) at Psomagen Inc. (Rockville, Maryland, USA) on an Illumina HiSeq6000 sequencer (Illumina, San Diego, California, USA).

The viral DNA from the four fecal samples of feral pigeons from the USA was amplified by rolling-circle amplification (RCA) using a TempliPhi 2000 Reaction Kit (GE Healthcare, Chicago, Illinois, USA). The RCA products of samples were used to prepare an Illumina sequencing library using the Illumina DNA library preparation (M) tagmentation kit (Illumina, San Diego, California, USA) and these were sequenced on an Illumina 4000 sequencer at Psomagen Inc. (Rockville, Maryland, USA).

The raw reads were trimmed with Trimmomatic-0.39 [26], and SPAdes version 3.12.0 assembly software [27] was used for de novo assembly of the sequencing reads into contigs. Contigs > 1000 nts were analyzed for viral-like sequences using NCBI Blast [28] against the Viral RefSeq protein database (release 210) downloaded from GenBank (NCBI).

### Bioinformatic analysis

Parvovirus-like contigs were subjected to BLAST search [28] to find related parvovirus sequences in GenBank database. The sequences were aligned with MAFFT method [29] in Geneious Prime v.2024.0.3 software (Dotmatics, Boston, Massachusetts, USA). Open reading frames (ORFs) and complete coding sequences (CDs) were identified with Find ORFs tool, also in Geneious Prime software, and the sequences were annotated. Because only a few pigeon parvovirus sequences are available in GenBank to date, in order to enhance the analysis, we also included two coding-complete genome sequences identified in feces of a feral pigeon collected in USA. The new annotated parvovirus sequences, two from USA and three from Poland, were deposited in the GenBank database under accession numbers PP965732, PP965733, PP965734, PP970575 and PP970576, while all metagenomic data can be found in the BioProject database with accession numbers PRJNA1187544 and PRJNA1045671 under BioSample accession numbers SAMN45493607, SAMN45493608 and SAMN45500220.

For phylogenetic analysis, regions coding the non-structural protein (NS) and structural protein (VP) were extracted, and NS sequences were translated to amino acids with Geneious Prime’s Translate tool (Dotmatics, Boston, Massachusetts). Pairwise identity matrices of NS amino acid sequences described in this study and acquired from GenBank were generated in SDT v1.3 software [30]. Subsequently, ‘Find DNA/protein’ models tool in MEGA 11 software [31] was used to find substitution models adequate for phylogenetic analysis of every set of aligned sequences. IQ-TREE 1.6.12 software [32, 33] was then used to perform maximum likelihood analysis with 1,000 bootstrap replicates, and iTOL v6 software [34] allowed us to visualize the phylogenetic trees.

### Statistical analysis

Chi-square test (χ^2^) was used to evaluate the correlation of the prevalence of pigeon parvovirus 1 with the health status of the pigeons, while non-parametric Mann–Whitney U test was used to evaluate the correlation of the amount of the pigeon parvovirus 1 genetic material in positive samples with the health status of the pigeons. Statistica 13 software (Statsoft, Cracow, Poland) was used to perform both analyses, and differences were considered significant with *p* < 0.05.

## Results

### Quantitative analysis

#### TaqMan qPCR

The specificity of the TaqMan qPCR assay was proven by negative results of all the tested viruses except for pigeon parvovirus 1. The sensitivity of the qPCR assay was determined as 3.018 copies of the amplicon/μl. The standard curve reflecting the results of the sensitivity test is illustrated in Additional file 1, and its characteristics are as follows: slope − 3.4458, efficiency 95%, error 0.23, R^2^ 1.00 and Y-intercept 34.96.

TaqMan qPCR revealed 29 samples out of 90 (32.2%) in group S and 30 samples out of 63 (47.6%) in group C to be positive for genetic material of pigeon parvovirus 1, and the difference between the groups was found non-significant with χ^2^ = 3.71 and *p* = 0.0542. 15 flocks out of 24 (62.5%) in group S and 12 flocks out of 13 (92.3%) in group C had pigeons testing positive. There was one flock in group S in which all the tested birds were positive.

#### ddPCR

**Fig. 1:**
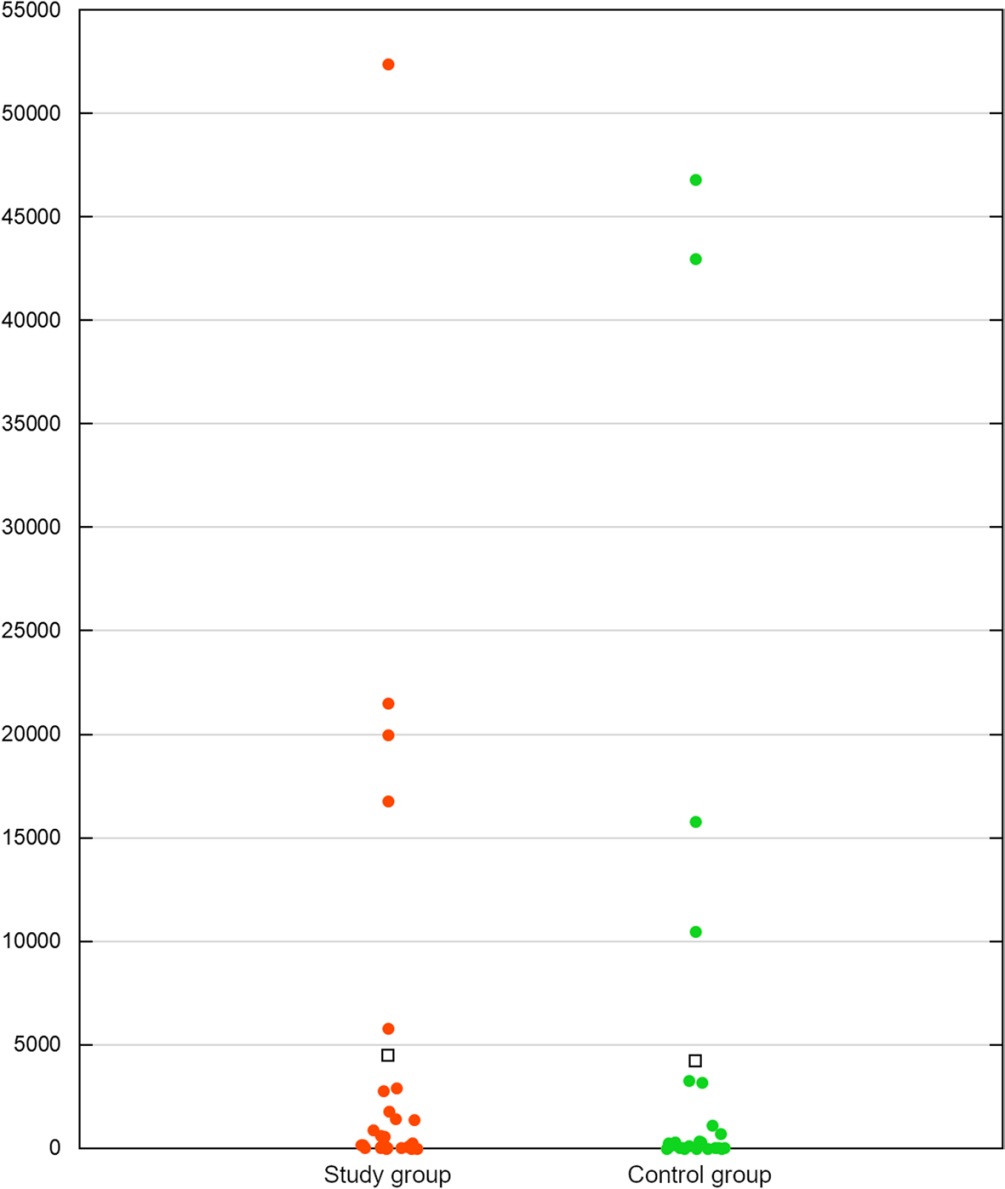
The graphical presentation of the results of ddPCR in samples testing positive for pigeon parvovirus genetic material in both study and control group, expressed as mean genome copy number per 20 μl of the sample. Blank squares represent the mean values in the two study groups. The difference in genome copy number between the groups was found non-significant in non-parametric Mann–Whitney U test with *p* = 0.1922

ddPCR revealed that the mean pigeon parvovirus 1 genome copy number in 20 µl of the samples measured 4,492.48 ± 10,961.92 in the S group and 4,208.63 ± 11,581.84 in the C group. The difference between the groups was also found non-significant with *p* = 0.1922. The results of ddPCR are illustrated in Fig. 1.

### Pairwise and phylogenetic analyses

Two coding-complete genome sequences of parvoviruses were determined through HTS of the swab samples from pigeons in Poland. The sequences, named PL_pigeon_11a/2023 (PP970575) and PL_pigeon_15a/2023 (PP970576), were 5,638 nt and 5,510 nt in length and had GC content of 51.9% and 48.7%, respectively. They both encoded four proteins: a non-structural protein (NS1), structural protein (VP) and two hypothetical proteins. The 5’ and 3’ ends of the two sequences had untranslated regions (UTRs). The BLAST search revealed that both newly obtained sequences appeared to belong to *Aveparvovirus* genus within the *Parvovirinae* subfamily. The pairwise analysis of the coding-complete genome sequences revealed 68.8% nucleotide identity between the two newly recovered *Aveparvovirus* strains from Poland. PL_pigeon_11a/2023 and PL_pigeon_15a/2023 shared respectively 92.8% and 69.1% nucleotide identity with pigeon parvovirus 1 strain HK8 (KC876004), a member of the species *Aveparvovirus columbid1*. Both sequences were detected in samples from control group. As for the two sequences identified in pigeon feces from USA, BLAST search revealed that USA_pigeon_K18.1.AV/2021 (PP965734) appeared to belong to *Chaphamaparvovirus* genus within the *Hamaparvovirinae* subfamily and USA_pigeon_K18.2.AV/2021 (PP965732) appeared to belong to to *Aveparvovirus* genus within the *Parvovirinae* subfamily. USA_pigeon_K18.2.AV/2021 had a genome organization similar to that of the described Polish strains—the sequence was 5,249 nt in length and its GC content was 51,4%, it encoded the same proteins and also had UTRs at 5’ and 3’ ends. In turn, marked differences can be seen in USA_pigeon_K18.1.AV/2021 – the sequence was shorter (4,371 nt) and of lower GC content (39,8%), and encoded one hypothetical protein, two non-structural proteins and one structural protein; it also had UTRs at 5’ and 3’ ends. The nucleotide identity between the two strains from USA measured 50.6%. USA_pigeon_K18.1.AV/2021 shared 82.7% nucleotide identity with pigeon-associated parvo-like virus isolate PLK23 (OR800330) and USA_pigeon_K18.2.AV/2021 shared 94.9% nucleotide identity with pigeon parvovirus 1 strain HK8 (KC876004). All the sequences with which the newly recovered sequences were compared were the most similar hits revealed by BLAST search and were acquired from GenBank database for pairwise analysis.

Additionally, a partial sequence of structural protein gene named PL_pigeon_K40/2023 was also obtained, and it received GenBank accession number PP965733. It was 1,858 nt in length and was incomplete at the 3’ end, consisting of approximately 88.9% of the length of this gene in pigeon parvovirus 1 strain HK8 sequence (KC876004). The BLAST search revealed that the partial sequence is also the most likely to belong to *Aveparvovirus* genus within the *Parvovirinae* subfamily. This sequence was also detected in a sample from control group.

**Fig. 2:**
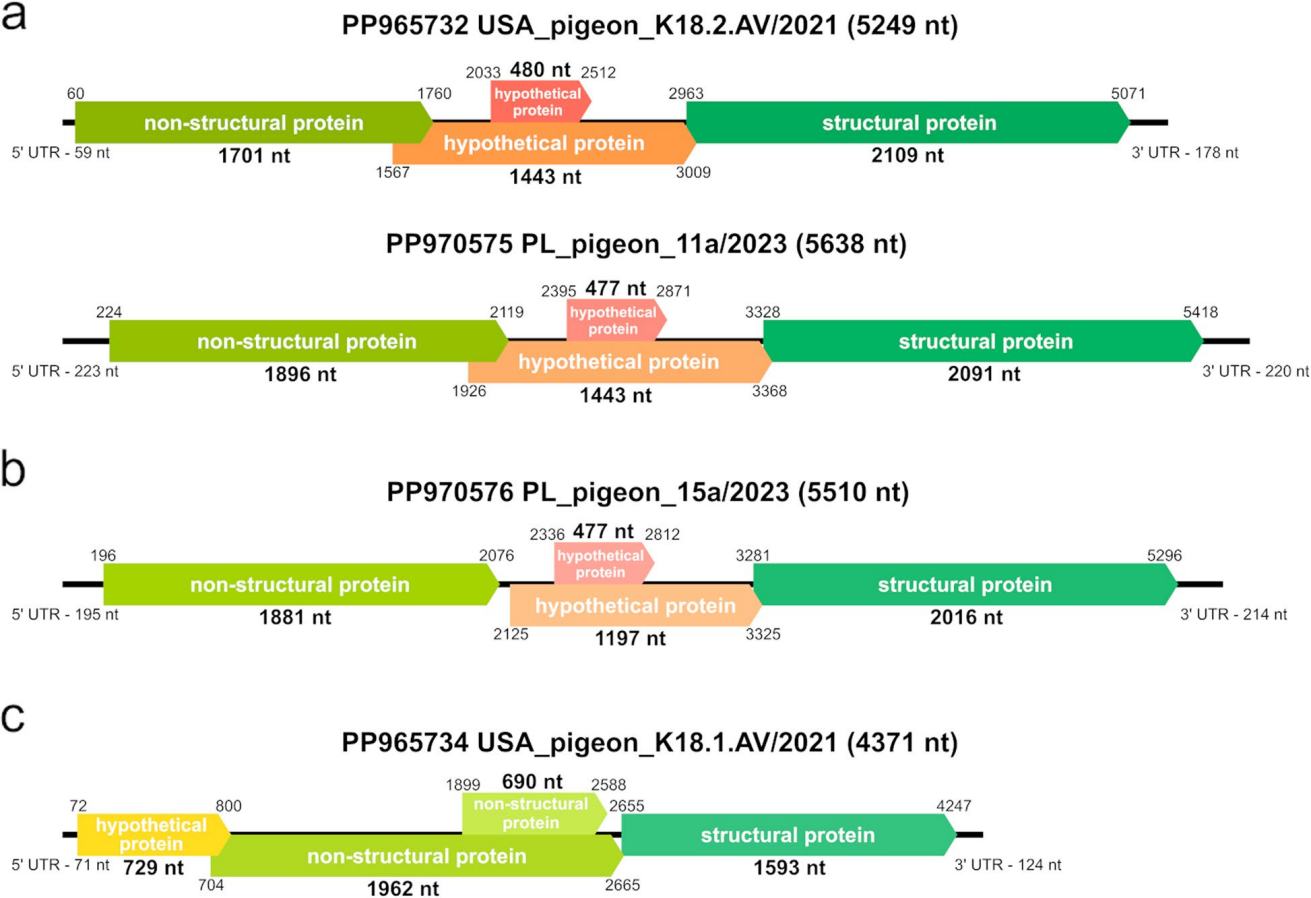
Schematic illustration of genome organization of coding-complete genome sequences of pigeon parvoviruses obtained in this study: **a** pigeon parvovirus 1 sequences from USA and Poland, **b** proposed pigeon parvovirus 2 sequence from Poland, and **c** pigeon chaphamaparvovirus sequence from USA

The genome organization of the coding-complete genomes originating from pigeons from Poland and USA is illustrated in Fig. 2.

**Fig. 3:**
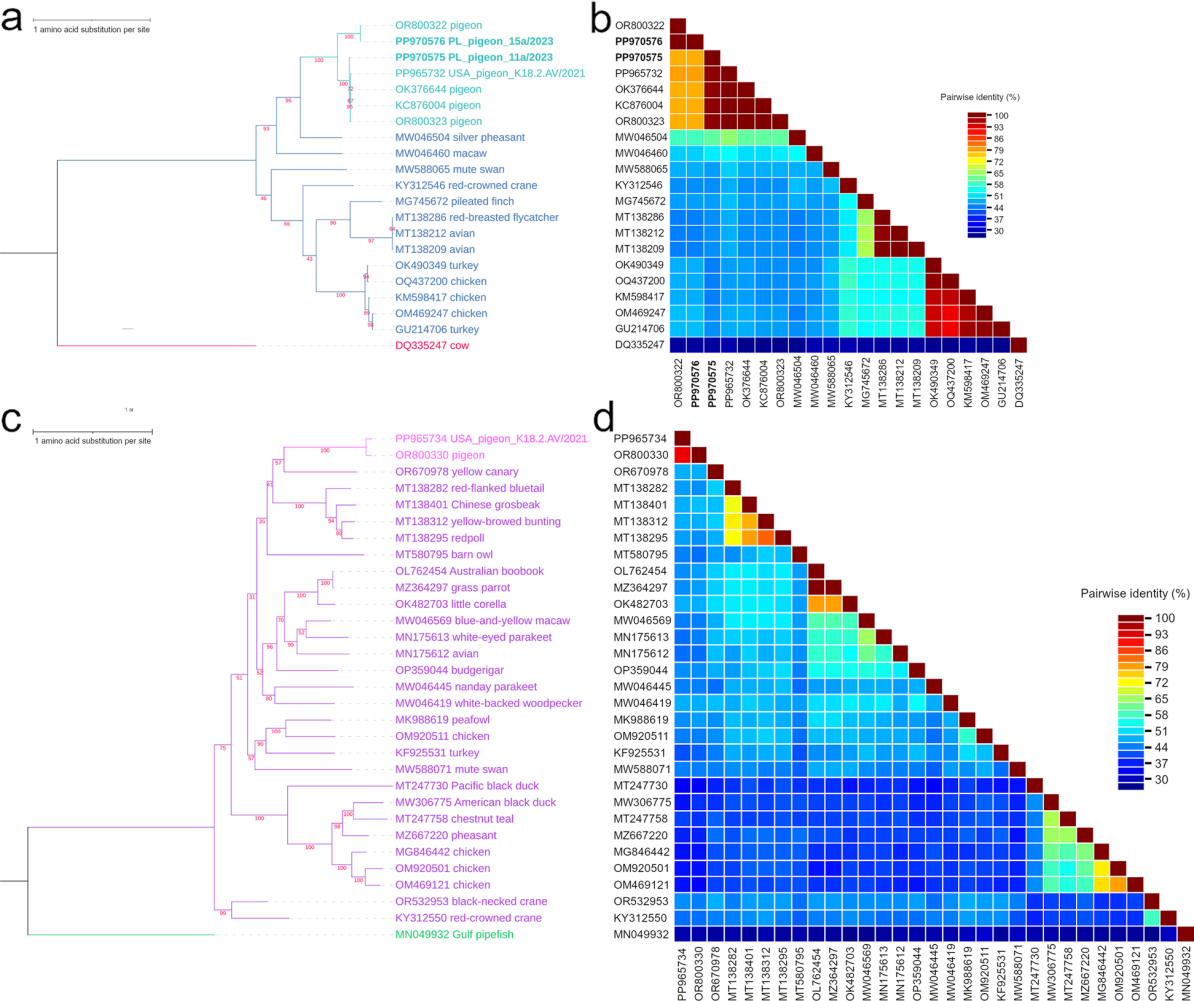
**a** Maximum likelihood phylogenetic tree made with LG + G substitution model with 1,000 bootstrap replicates, composed of amino acid sequences of NS1 of aveparvoviruses described in this study along with those acquired from GenBank database. The novel sequences described in this study are labeled with accession number and strain name, with pigeon aveparvoviruses from Poland written in bold, while the rest of the sequences is labeled with accession number and host name. The pigeon aveparvoviruses are colored with turquoise and the other aveparvovirus sequences are colored with navy blue. The tree is rooted with a sequence of bovine parvovirus 1 of *Bocaparvovirus* genus and this sequence is colored with red. **b** Pairwise identity matrix of amino acid sequences of non-structural protein (NS1) of aveparvoviruses described in this study along with those acquired from GenBank database. All the sequences are labeled with accession number, and accession numbers of pigeon parvoviruses from Poland are written in bold. The amino acid identity of every pair of sequences is represented on a matrix by a color corresponding to the percentage value – from dark blue representing the lowest identity percentage to dark red representing the highest identity percentage. **c** Maximum likelihood phylogenetic tree made with LG + G + I + F substitution model with 1,000 bootstrap replicates, composed of amino acid sequence of NS1 of pigeon chaphamaparvovirus described in this study along with avian chaphamaparvoviruses acquired from GenBank database. The novel sequence described in this study is labeled with accession number and strain name, while the rest of the sequences is labeled with accession number and host name. The pigeon chaphamaparvoviruses are colored with pink and the other chaphamaparvovirus sequences are colored with purple. The tree is rooted with a sequence of *Syngnathus scovelli* chapparvovirus of *Ichtchaphamaparvovirus* genus and this sequence is colored with green. **d** Pairwise identity matrix of amino acid sequences of non-structural protein (NS1) of pigeon chaphamaparvovirus described in this study along with avian chaphamaparvoviruses acquired from GenBank database. All the sequences are labeled with accession number. The amino acid identity of every pair of sequences is represented on a matrix by a color corresponding to the percentage value – from dark blue representing the lowest identity percentage to dark red representing the highest identity percentage

Figure 3a shows a maximum likelihood phylogenetic tree constructed of amino acid sequences of NS1 of aveparvoviruses described in this study and acquired from GenBank database. The tree was inferred with LG + G substitution model. The two pigeon parvovirus sequences from Poland and USA_pigeon_K18.2.AV/2021 sequence form a common cluster with sequences acquired from pigeons available in GenBank. Within this cluster two smaller subclusters can be recognized. One is formed by PL_pigeon_11a/2023 (PP970575), K18.2.AV/2021 (PP965732) and representants of *Aveparvovirus columbid1* species: HK8 (KC876004), FJ01 (OK376644) and PiPV/PLK/2023 isolates (OR800323). The other subcluster is formed by PL_pigeon_15a/2023 (PP970576) and another isolate also named PiPV/PLK/2023 (OR800322). Sequences acquired from silver pheasant (MW046504) and macaw (MW046460) form the basal branches of the pigeon cluster; and sequences of poultry, mute swan, red-crowned crane, pileated finch, red-breasted flycatcher, and unknown avian species form another separate cluster within the *Aveparvovirus* group.

In Fig. 3b, pairwise identity matrix of the same sequences can be seen. The two sequences acquired from pigeons from Poland share 78.1% amino acid identity within the NS1. PL_pigeon_11a/2023 (PP970575) shares 78.1% and 98.8% average amino acid identity with feral pigeon parvovirus A isolate PiPV/PLK/2023 (OR800322) and the rest of the pigeon aveparvovirus sequences, respectively. In turn, PL_pigeon_15a/2023 (PP970576) shares 100% and 77.9% average amino acid identity with OR800322 and the rest of the pigeon aveparvovirus sequences, respectively.

Figure 3c presents a maximum likelihood phylogenetic tree inferred with LG + G + I + F substitution model and constructed of amino acid sequences of NS1 of avian chaphamaparvoviruses. USA_pigeon_K18.1.AV/2021 (PP965734) forms a branch together with pigeon-associated parvo-like virus isolate PLK23 (OR800330) in a cluster formed by passerine and barn owl sequences. Multiple different sequences acquired from wild birds and poultry form different branches in the tree, and as it can be seen in Fig. 3d, most of them share low amino acid identities with USA_pigeon_K18.1.AV/2021. The chaphamaparvovirus sequence from USA shares 91.4% and 42.5% average amino acid identity within the NS1 with its closest relative, PLK23 isolate (OR800330) and other avian chaphamaparvovirus sequences, respectively.

**Fig. 4:**
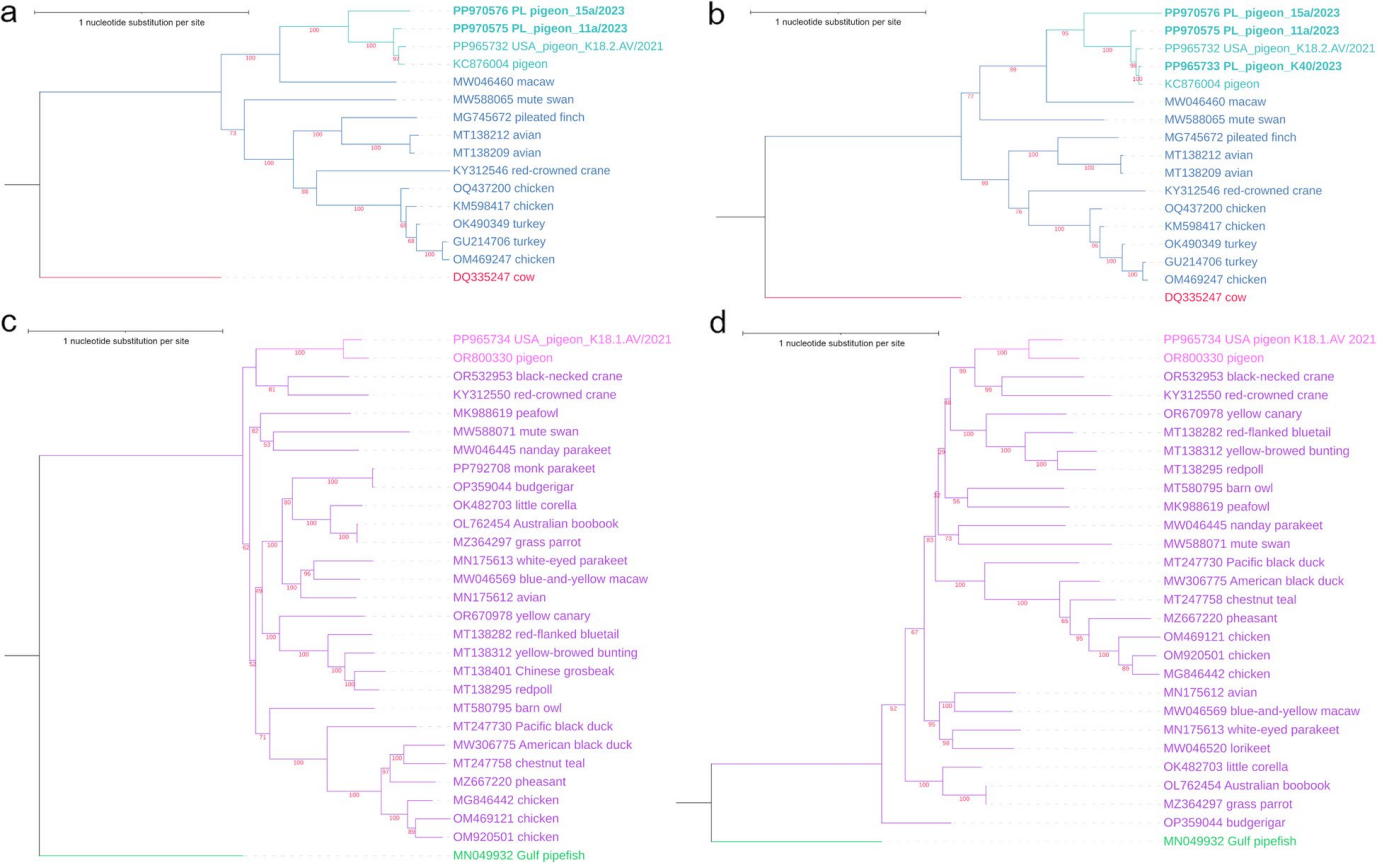
Maximum likelihood phylogenetic trees made with GTR + G + I substitution model, composed of pigeon parvovirus sequences described in this study along with other representants of *Parvoviridae* family acquired from GenBank database: **a** tree of complete genomic sequences of aveparvoviruses, **b** tree of VP gene sequences of aveparvoviruses, **c** tree of complete genomic sequences of avian chaphamaparvoviruses, **d** tree of VP gene sequences of avian chaphamaparvoviruses. The number of bootstrap replicates is 1,000. Both aveparvovirus trees are rooted with a sequence of bovine parvovirus 1 of *Bocaparvovirus* genus, while both chaphamaparvovirus trees are rooted with a sequence of *Syngnathus scovelli* chapparvovirus of *Ichtchaphamaparvovirus* genus. The sequences described in this study are labeled with accession number and strain name, with pigeon parvoviruses from Poland written in bold, while the rest of the sequences is labeled with accession number and host name. The aveparvovirus sequences are colored with navy blue with the exception of pigeon aveparvoviruses colored with turquoise, and the bocaparvovirus sequence is colored with red. In turn, the chaphamaparvovirus sequences are colored with purple with the exception of pigeon chaphamaparoviruses colored with pink, and the ichtchaphamaparvovirus sequence is colored with green

In turn, maximum likelihood phylogenetic trees using GTR + G + I substitution model and inferred from the aligned nucleotide sequences of parvoviruses are shown in Fig. 4. Genetic relationship of the complete genome sequences and VP gene sequences of aveparvoviruses can be seen in Fig. 4a and b, respectively. Both trees have similar structure, with the newly determined sequences from pigeons from Poland clustering together with USA_pigeon_K18.2.AV/2021 (PP965732) and with the only available complete genomic sequence of pigeon parvovirus A strain HK8 (KC876004) in the species *Aveparvovirus columbid1*, and it can be noted that PL_pigeon_15a/2023 is basal to other members of the species. In both trees, sequence acquired from macaw again forms the basal branch of the pigeon cluster; and sequences of poultry, mute swan, pileated finch and unknown avian species form a separate cluster. As for the avian chaphamaparvovirus complete genome sequences and VP gene sequences arranged in trees in Fig. 4c and d, respectively, it can be observed that, similarly to the tree of NS1 amino acid sequences, USA_pigeon_K18.1.AV/2021 (PP965734) forms branches together with pigeon-associated parvo-like virus isolate PLK23 (OR800330); although the sequences acquired from cranes are the most related with them in both trees of question.

## Discussion

Viral enteropathies, which affect mostly young pigeons during their first trainings and races, are a common health problem in rearing of racing pigeons. Diseases of digestive tract lead to reduced absorption of nutrients, resulting in low body condition and poor performance in races. Similarly to viral enteropathies commonly noted in domestic poultry, several viruses including parvoviruses may be involved in this problem [13, 35–37]. Viruses in the *Parvoviridae* family are known to take part in multifactorial disease syndromes, and because different species and strains may vary in pathogenicity and cell tropism, their role in the occurrence and course of enteric disease of pigeons might be overlooked [7, 13]. In this study we made attempts to seek a connection between the detection of pigeon parvovirus genetic material and presence of enteropathy in young racing pigeons.

The quantitative analysis of pigeon parvovirus 1 DNA in the tested samples yielded unexpected results. The virus was found to be noticeably more prevalent in the asymptomatic control group than in the clinically diseased study group, although the difference was found to be statistically insignificant. Viral load analyses of pigeon parvovirus 1 in the samples showed the mean number of genome copies turned out to be very similar in the two groups. Positive samples acquired from pigeons from both groups varied considerably in the amount of the detected genetic material of the tested virus, which can be clearly observed in Fig. 2. These observations do not allow us to draw any definitive conclusions regarding the connection between the presence of pigeon parvovirus 1 DNA and the occurrence of enteric disease in pigeons, because in our study pigeon parvovirus 1 was prevalent in both clinically affected and healthy birds with its occurrence more often in the asymptomatic group. It is also worth mentioning that all parvovirus sequences recovered by HTS were derived from samples from the asymptomatic control group. The reason for such situation may be either non-pathogenicity or low pathogenicity of the detected pigeon parvovirus strains; according to Tijssen et al. [1], some parvoviruses require co-infection with other viruses, for example adenoviruses or herpesviruses, to cause clinical signs. Unfortunately, it is difficult to prove in this case because virtually nothing is known about the pathogenicity of parvoviruses in pigeons. To date there is only one paper available describing findings of parvoviruses in these birds, and it involves screening of fecal samples originating from feral pigeons of unknown health status [12]. There are reports concerning findings of parvovirus-like structures in electron microscopy of pigeon hepatocytes; however, no attempts to isolate the virus from tissues have been made and no information on the health status of the birds are available, either [38, 39]. Parvoviruses are known to be associated with occurrence of diarrhea in multiple animal species, with classical enteropathy affecting i.e. dogs and cattle, especially young individuals with not yet fully developed immune system [4, 7, 8, 40]. It is similar in case of birds, as many avian species are also prone to parvoviral infection, which may present differently depending on age of the host [13, 15, 23, 41, 42]. Both the pairwise identities and the position of the pigeon parvoviruses in the inferred phylogenetic trees suggest that the newly determined sequences are genetically distant from potentially pathogenic poultry parvoviruses, and there is no information on health status on either pigeons or wild bird species being the source of more genetically similar sequences. The results of the study described here imply that more research would be required to explore the link between pigeon parvoviruses and enteropathy. The results of such research could be meaningful, especially considering the high prevalence of pigeon parvovirus 1 noted in domestic pigeons in this study, contrasting with the lack of research in pigeons. Taking possible simultaneous infection with other viruses into account might be the key to elaborate on potential pathogenic effect caused by pigeon parvoviruses.

While quantitative analysis provided little new insight into the pathogenicity of parvoviruses to pigeons, the results of HTS were undoubtedly interesting. This method allowed us to determine and then identify two coding-complete genome sequences and one partial VP sequence of pigeon parvovirus from the samples from Poland, as well as two coding-complete genome sequences from the USA. This finding is especially compelling considering that up until now there was only one complete genomic sequence and eight partial sequences of pigeon parvovirus available in GenBank. It is also worth mentioning that all available sequences originate from either China or Thailand, meaning that this study is the first report of parvoviruses in pigeons outside Asia. While parvoviruses of poultry has been reported in Poland multiple times [37, 43–45] and several reports on findings of parvoviruses in other bird species in European countries have also been made [14, 16, 17, 46], the lack of reports regarding pigeons in Europe is noticeable. Therefore, a potential exploration of geographical distribution and epidemiology of pigeon parvoviruses and other related aveparvoviruses is so far severely limited. However, we made every effort to compare the newly determined sequences to those available. Analyses of the genome organization and phylogeny of parvoviruses show that the NS is the most conserved gene among members of *Parvoviridae* family, and thus amino acid sequences of the protein encoded by this fragment are used to differentiate members of both genera and species within the family. A pairwise identity of > 85% amino acid identity in NS1 is used for species demarcation [2]. Following this principle, PL_pigeon_11a/2023 (PP970575) and USA_pigeon_K18.2.AV/2021 (PP965732), which share respectively 98.7% and 99.8% amino acid identity in NS1 with pigeon parvovirus 1 strain HK8 (KC876004), are members of the species *Aveparvovirus columbid1*. However, PL_pigeon_15a/2023 (PP970576) with 77.9% average amino acid identity with representative sequences of the species *Aveparvovirus columbid1*, likely represents new species. The distant relationship of PL_pigeon_15a/2023 (PP970576) with members of the *Aveparvovirus columbid1* species is further supported by its localization in the obtained phylogenetic trees. Therefore, we initially name PL_pigeon_15a/2023 (PP970576) pigeon parvovirus 2, representing new species in the *Aveparvovirus* genus. Finally, one more interesting finding described here is the determination of USA_pigeon_K18.1.AV/2021 sequence (PP965734) which appears to be a representative of *Chaphamaparvovirus* genus within the *Hamaparvovirinae* subfamily. This strain probably belongs to the same species as isolate PLK23 (OR800330), previously assigned as pigeon-associated parvo-like virus, based on NS1 amino acid identity of 91.4%. The newly described sequence is genetically distant from the other known avian chaphamaparvovirus sequences, sharing an average 42.5% amino acid identity in NS1 with them, and seems to be the most closely related to sequences originating from passerine species. This paper would be the first description of detection of a member of *Hamaparvovirinae* subfamily in pigeons.

## Conclusions

In this study, we describe the determination of two coding-complete pigeon parvovirus genomes from Poland and two from USA. PL_pigeon_11a/2023 (PP970575) and USA_pigeon_K18.2.AV/2021 (PP965732) are most closely related to members of *Aveparvovirus columbid1* species. PL_pigeon_15a/2023 (PP970576) strain from Poland presumably represents new species. USA_pigeon_K18.1.AV/2021 (PP965734) strain from the USA, together with pigeon-associated parvo-like virus isolate PLK23 (OR800330) identified in Thailand, likely represent the same species in *Chaphamaparvovirus* genus within the *Hamaparvovirinae* subfamily. The results of quantitative analysis carried out within the frameworks of this study indicate that it is not yet clear what role, if any, parvoviruses play in the occurrence of enteric disease in pigeons. The findings of our research emphasize the need to further explore the poorly understood topic of pigeon parvoviruses.

## Supplementary Information

Additional file 1. The graphical presentation of the results of the TaqMan qPCR sensitivity test: standard curve (a) and amplification curves (b). The characteristics of the standard curve are as follows: slope − 3.4458, efficiency 95%, error 0.23, R^2^ 1.00 and Y-intercept 34.96. The amplification curves represent subsequent decimal dilutions (3.018 × 10^7^ to 3.010) with every reaction performed in triplicate.

## Data Availability

The data that support the findings of this study are available in the repository of University of Warmia and Mazury in Olsztyn, Poland at the link 10.31648/UWM371b5af78c1a4b9c89c8ce3146d1e490. The nucleotide sequence data obtained in this study were deposited in GenBank database under following accession numbers: PP970575, PP970576, PP965732, PP965733, PP965734, while all metagenomic data can be found in the BioProject database with accession numbers PRJNA1187544 and PRJNA1045671 under BioSample accession numbers SAMN45493607, SAMN45493608 and SAMN45500220.
