# Supplementary figures and images for: Known and novel parvoviruses identified in domestic pigeons
Source: BMC Vet Res. 2025 Jan 31;21:47. doi: 10.1186/s12917-025-04510-8 (PMC11783907; doi:10.1186/s12917-025-04510-8)

**a**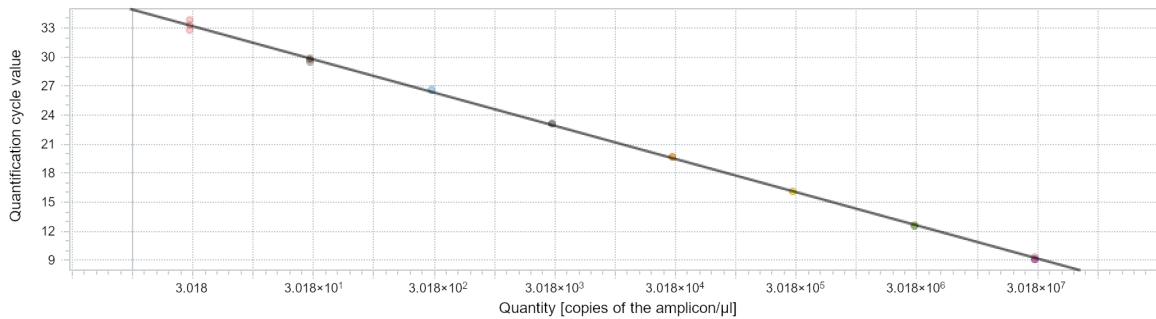**b**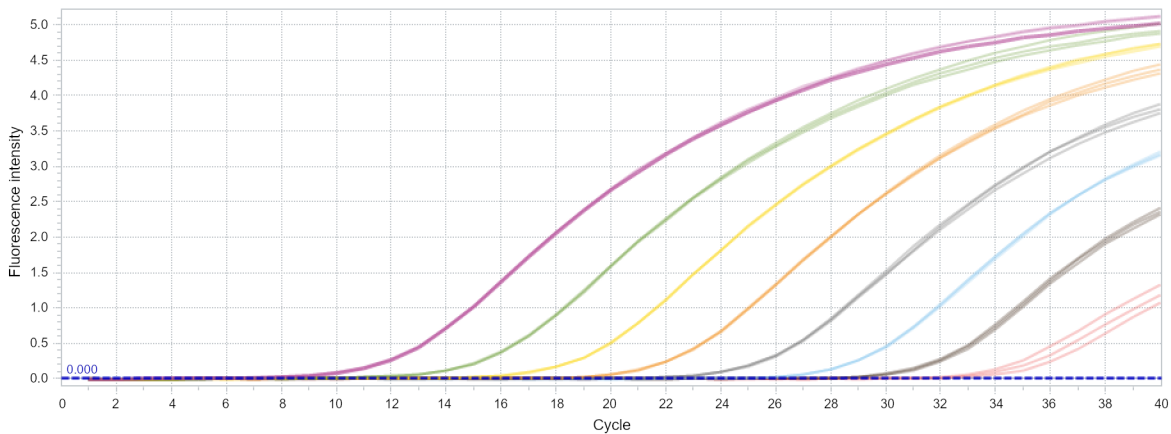

Supplement: Supplementary file 1 — Additional file 1. The graphical presentation of the results of the TaqMan qPCR sensitivity test: standard curve (a) and amplification curves (b). The characteristics of the standard curve are as follows: slope − 3.4458, efficiency 95%, error 0.23, R2 1.00 and Y-intercept 34.96. The amplification curves represent subsequent decimal dilutions (3.018 × 10^7^ to 3.010) with every reaction performed in triplicate. [file 12917_2025_4510_MOESM1_ESM.pdf]
